# Incidence and Outcome of Early Acute Kidney Injury in Critically-Ill Trauma Patients

**DOI:** 10.1371/journal.pone.0077376

**Published:** 2013-10-17

**Authors:** Amber S. Podoll, Rosemary Kozar, John B. Holcomb, Kevin W. Finkel

**Affiliations:** 1 Department of Internal Medicine, Division of Renal Diseases and Hypertension, Section of Critical Care Nephrology, University of Texas (UT) Health Science Center at Houston, Medical School, Houston, Texas, United States of America; 2 Department of Surgery, Division of Acute Care Surgery, Center for Translational Injury Research, Trauma and Critical Care Section, UTHealth Science Center at Houston, Medical School, Houston, Texas, United States of America; 3 Texas Trauma Institute, Memorial Hermann Hospital-Texas Medical Center, UTHealth Science Center at Houston, Medical School, Houston, Texas, United States of America; University of Pittsburgh Medical Center, United States of America

## Abstract

**Objective:**

To determine the incidence and effect on mortality of early acute kidney injury in severely injured trauma patients using the Acute Kidney Injury Network creatinine criteria.

**Design:**

A retrospective cohort study of severely injured trauma patients admitted to the shock trauma intensive care unit.

**Setting:**

Texas Trauma Institute, a state designated level I trauma unit certified by the American College of Surgeons Committee on Trauma.

**Patients:**

901 severely injured trauma patients admitted over a 15 month period to the shock trauma intensive care unit.

**Interventions:**

Retrospective analysis of prospectively collected data abstracted from an electronic trauma database.

**Measurements and Main Results:**

Of 901 eligible patients admitted to the shock trauma intensive care unit after traumatic injury, 54 patients (6%) developed acute kidney injury, of whom 10 (19%) required renal replacement therapy. The 30-day mortality rate for the entire cohort was 83/901 (9.2%). Patients with early acute kidney injury had a mortality rate of 16/54 (29.6%). When corrected for multiple covariates including injury severity scores, the development of early acute kidney injury was associated with a significantly higher risk of death at 30 days with an OR of 3.4 (95% CI 1.6-7.4).

**Conclusions:**

Applying the Acute Kidney Injury Network creatinine criteria in severely injured trauma patients, the incidence of early acute kidney injury was 6%. After correction for injury severity, development of early acute kidney injury was independently associated with significantly higher 30-day mortality.

## Introduction

The development of acute kidney injury (AKI) in critically ill patients is associated with increased morbidity and mortality [1], [2], [3]. In a large multinational, multicenter observational study involving over 29,000 critically ill patients over a 16-month period, the prevalence of severe AKI was 5.7% and the need for renal replacement therapy was 4.3% [4]. Overall hospital mortality of AKI patients was 60.3%. Of surviving AKI patients, 13.8% required dialysis at the time of discharge.

Major trauma accounts for 15% of intensive care unit admissions annually in the United States and is the leading cause of death in people younger than 45 years of age. In critically ill patients with traumatic injury, the impact of developing AKI has been previously described using varying definitions. Brandt, et al found that a rise in creatinine to a level above 1.5 mg/dL, an increase in baseline creatinine of >50%, or an absolute increase of creatinine of more than 0.5 mg/dL conferred a seven-fold increase in mortality [5]. It also increased hospital length of stay and average cost per case. In a large multicenter study involving 72,757 trauma patients in nine centers, using a definition of an increase in creatinine of 2.0 mg/dL over admission levels, the development of AKI resulted in a significantly increased mortality rate, particularly in those that required renal replacement therapy [6].

Weaknesses in previous studies are the lack of a consensus definition of AKI and a uniform assessment of injury severity. The Acute Dialysis Quality Initiative group developed a definition for AKI based on RIFLE criteria [7]. They classified AKI into 3 degrees of severity based on incremental increases in creatinine levels or decreases in urine output (Risk, Injury, and Failure), and 2 clinical outcomes (Loss and End-stage renal disease). Several studies have shown that the RIFLE criteria are an independent predictor of outcome in critically ill patients with AKI [8], [9], [10], [11]. Using the RIFLE classification in a multicenter retrospective analysis of more than 9,000 critically ill trauma patients in 57 centers, early (within 24 hours of admission) AKI was independently associated with increased rate of hospital mortality [12]. New guidelines for the definition of AKI have been proposed by the Acute Kidney Injury Network (AKIN) based on a modification of the RIFLE criteria (Table 1) [13]. We hypothesized that critically ill patients with traumatic injury who develop AKI as defined by the AKIN creatinine criteria would have significantly increased 30-day mortality rates after controlling for injury severity.

**Table 1:** AKIN AKI Criteria.

Stage	Creatinine Criteria	Urine Output Criteria
1	Increase in serum creatinine of ≥0.3 mg/dL or increase of ≥50–200% (1.5 to 2-fold) increase above baseline	<0.5 mL/kg/hr for >6 hours
2	Increase in serum creatinine of >200–300% (>2 to 3-fold) above baseline	<0.5 mL/kg/hr for >12 hours
3	Increase in serum creatinine of >300% (>3-fold) above baseline or a serum creatinine ≥4 mg/dL with an acute rise of ≥0.5 mg/dL	<0.3 mL/kg/hr for 24 hours or anuria for 12 hours

AKIN: Acute Kidney Injury Network.

AKI: Acute Kidney Injury.

Adapted from reference 13.

## Materials and Methods

The Institutional Committee for the Protection of Human Subjects issued a formal written waiver of approval of authorization and need for ethics approval to access de-identified data from a Trauma Registry prior to beginning this study. Written informed consent for initial patient data entry was unnecessary because it is a Trauma Registry required by law for all trauma centers.

### Study Design

This was a retrospective analysis of prospectively collected data using a trauma database of several thousand patients treated at a single regional trauma center. Adult (≥18 years) patients admitted to the shock trauma intensive care unit from January 1, 2009 to March 1, 2010 composed the study cohort. The Texas Trauma Institute, located in Houston, Texas (metropolitan area 6 million residents), is a state designated level I trauma center verified by the American College of Surgeons Committee on Trauma and operates the busiest ambulance heliport in the United States.

All patients had been directly transferred from the site of trauma to the hospital. Patients with isolated head injury and burns were excluded from the study because they were admitted to separate dedicated units and do not contribute to the trauma data base. The majority of patients (95%) had multiple injuries from motor vehicle accidents.

Data collected at the time of arrival to the hospital included demographics, mechanism of injury, Glasgow Coma Scale score, emergency department vital signs, time from injury to emergency center arrival, and laboratory parameters. Severity of trauma was measured by calculation of the Injury Severity Score from the Abbreviated Injury Scale [14]. The score is an anatomical scoring system that provides an overall score for patients with multiple injuries. Six organ systems are scored on the 0–6 scale. The 3 worse scores are squared and added. The range is 0–75. Any score of 6 (non-survivable injury) results in a score of 75.

Patients were classified as early AKI (<72 hours from time of injury) by AKIN creatinine criteria. The lowest creatinine level in the first 24-hours of admission was used for determining the change in creatinine. Patients were excluded if they had a known history of chronic kidney or on chronic dialysis. Chronic kidney disease was defined as an estimated glomerular filtration rate less than 60 ml/minute based on the National Kidney Foundation K/DOQI clinical practice guidelines. Urine output was inconsistently recorded and was not used for defining AKI. Outcomes measured were incidence of early AKI, need for renal replacement therapy, and 30-day mortality.

### Statistical Analysis

Analysis was performed using SAS 9.2 (SAS Institute Inc., Cary, NC, USA). In the event of missing data values, data were not replaced. Normally or near normally distributed variables are reported as means with standard deviations (SD) and compared by Student's t-test. Non-normally distributed continuous data are reported as medians with inter-quartile ranges (IQR) and compared by Mann Whitney U test. Categorical data were reported as proportions and compared using Fisher's Exact Test. Multivariate logistic regression analysis was used to assess the association of AKI with hospital mortality, adjusting for age, gender, injury severity score, diabetes and hypertension. Model fit was assessed by the goodness-of-fit test. Data are presented as odds ratios (OR) with 95% confidence intervals (CI). A *p* value of <0.05 was considered statistically significant for all comparisons.

## Results

Over a 15 month period, 915 critically ill patients were admitted to the shock trauma intensive care unit. We excluded 14 patients with chronic kidney disease, or missing mortality or renal replacement therapy information, ending with a total of 901 patients. Baseline demographics are shown in Table 2. The mean age was 42 years and patients were predominantly male (73%). The mean Injury Severity Score was 23.4 (SD 13.1) indicating a severely injured population. By AKIN creatinine criteria, 54 patients (6%) developed early AKI, with stage 1 (85%) being much more common than either stage 2 (11%) or stage 3 (4%). Patients who developed early AKI were older and had higher baseline creatinine levels than those without early AKI. There was no statistically significant difference in gender, Glasgow Coma Scale score, emergency department mean blood pressure, time of injury to emergency center arrival, diabetes, or hypertension between patients with or without early AKI. Patients who developed early AKI had higher abdominal Abbreviated Injury Scores. However, there was no significant difference between the groups' overall degree of injury based on the Injury Severity Score.

**Table 2:** Baseline characteristics.

Characteristics	No Early AKI (N = 847)	Early AKI(N = 54)	p-value
Age, in years [median (IQR)]	39 (26–52)	42 (26–57)	0.4
Age >55 (%)	20	33	0.04
Male gender (%)	72	83	0.08
Injury Severity Score [mean (SD)]	23 (13)	26 (14)	0.5
Time from injury to emergency room arrival [mean (SD)] (in minutes)	78 (15)	79 (17)	0.4
Baseline creatinine [median (IQR)] (in mg/dL)*	1.1 (0.9–1.3)	1.4 (1.0–1.6)	<0.0001
Glasgow coma score [median (IQR)]	15 (3–15)	14 (3–15)	0.3
Mean blood pressure [mean (SD)] (in mm Hg)	124 (35)	115 (38)	0.4
Diabetes mellitus (%)	5.2%	7.4%	0.5
Hypertension (%)	6.0	7.4	0.6
**Maximum head AIS (%)**			0.2
1–2	12	0	
3	41	28	
4	31	39	
5	16	33	
**Maximum Chest AIS (%)**			0.05
1–2	11	13	
3	36	29	
4	40	31	
5	13	27	
**Maximum ABD AIS (%)**			0.002
1–2	50	39	
3	28	30	
4	17	8	
5	5	19	

AKI: Acute kidney injury; IQR: Inter-quartile range; SD: Standard deviation; ED: Emergency department;

AIS: Abbreviated injury score.

* Lowest creatinine within 24 hours.

In the 54 early AKI patients, dialysis was required in 10 (19%) patients. The crude 30-day mortality rate for the entire cohort was 83/901 (9.2%). Patients who developed early AKI had a higher 30-day mortality rate of 16/54 (29.6%) [Table 3].

**Table 3:** Crude mortality rates.

All patients (%)	No Early AKI	Early AKI	Early AKI no RRT	Early AKI + RRT
83/901 (9.2%)	67/847 (7.9%)	16/54 (29.6%)	11/44(25%)	5/10 (50%)

AKI: Acute Kidney Injury; RRT: Renal replacement therapy.

The development of early AKI was associated with a significantly higher risk of death at 30-days with crude OR of 4.92 (95% CI 2.60- 9.28). After adjustment for age, gender and Injury Severity Score, early AKI remained independently associated with mortality with an adjusted OR of 3.41 (95% CI 1.57-7.40) [Table 4]. The need for renal replacement was not statistically associated with an increased mortality rate after adjustment for early AKI, age, gender and Injury Severity Score.

**Table 4:** Covariate adjusted mortality rates.

Characteristic	Crude OR (95% CI)	Adjusted OR (95% CI)
Early AKI	4.79 (2.54– 9.02)	3.41(1.57– 7.40)
Early AKI + RRT	10.45 (2.96– 36.90)	2.95 (0.58– 14.92)
Age >55	2.17 (1.34– 3.51)	2.60 (1.51– 4.45)
Male Gender	0.84 (0.51– 1.37)	0.97 (0.57– 1.66)
ISS	1.06 (1.04– 1.08)	1.07(1.05–1.09)

OR: Odds ratio; CI: Confidence intervals; AKI: Acute Kidney Injury; RRT: Renal replacement therapy;

ISS: Injury Severity Score.

## Discussion

We conducted a retrospective analysis of 901 critically ill patients admitted to a busy regional trauma center over a 15-month period to evaluate the outcomes of early AKI (occurring within 72-hours of admission) using the AKIN creatinine criteria. We found that early AKI occurred in 6% of patients admitted to the shock trauma intensive care unit. These patients were relatively young and predominantly male (73%). Early AKI was associated with both crude and covariate adjusted increases in hospital mortality. There was no statistical evidence that renal replacement therapy further increased 30-day mortality, which is likely explained by the small number of this subgroup (10 patients). In this study the incidence of early AKI in trauma was somewhat lower than generally reported in prior investigations despite the use of a more sensitive definition. For example, in a multi-center study from Finland, Ala-Kokko et al. described evidence of “renal dysfunction” in 10.8% and “renal failure” in 3.5% within 24 hours in a cohort of 1,044 patients with trauma [15]. Renal dysfunction and failure were classified by sequential organ assessment score. Brandt et al. found that “renal failure” occurred in 23.8% of patients when defined by an absolute serum creatinine >1.5 mg/dL or relative changes of >50% or >0.5 mg/dL [5]. Our incidence of early AKI was also lower than that reported by Bagshaw et al. using the RIFLE criteria in a total of 9449 trauma patients from 57 intensive care units across Australia [12]. In that study the incidence of early AKI (the first 24 hours) was 18.1%. There are a few plausible explanations for this discrepancy. First, our patients were generally younger and less likely to have co-morbid diseases. Second, because we are the major regional trauma center in the metropolitan area, the post-traumatic resuscitation is highly protocolized and transport time from the injury scene is minimized by the use of an air-ambulance service, whereas the Bagshaw data were derived from querying a larger database of all patients admitted to 57 Australian intensive care units comprising tertiary care, metropolitan, regional/rural, and private hospitals. This hypothesis is supported by the findings reported by Wohlauer et al [16]. These authors queried a 17-year old data base from another single center state designated level I trauma center and found the incidence of early AKI, defined as a creatinine greater than 1.8 mg/dl within 48 hours was 2.13%. Similar to our findings, the development of early AKI was associated with a 27% mortality rate.

Another strength of this study was controlling for injury severity by covariate adjustment for Injury Severity Score, an established anatomical score for comparing patients with multiple trauma.

There are limitations to our study. We report our results from a single center which can result in selection bias. It is also a highly select population of level I trauma patients with multiple injuries from car crashes. Therefore it is important to recognize that our findings may not be applicable to patients with other types of injury or those not referred to a level I trauma center. Finally, the diagnosis of AKI was based only on the creatinine criteria of the AKIN classification. However, it is uncertain whether urine output and creatinine criteria are of equivalent utility. Studies suggest that urine output is a more sensitive marker of AKI but has a less pronounced impact on mortality compared to creatinine increases [10]. This omission could have led to a lower incidence of AKI.

Our study is retrospective in nature. However, the data were prospectively collected in an established trauma database at the second busiest trauma center in the United States. Given the nature of the database, it was not possible to control for several factors including contrast administration, degree of blood loss, intra-abdominal hypertension, or volume resuscitation. However, these confounders are risk factors for the development of AKI and do not necessarily negate the direct impact of AKI itself on mortality. This contention is supported by the similarity of the Injury Severity Scores between the groups with and without AKI.

Finally, we only considered the development of early AKI (the first 72 hours) rather than the development of AKI later in the cohort's hospital course. We chose this time period specifically to avoid confounding factors such as late onset sepsis or nephrotoxic injury. Also, patients were not analyzed separately by the severity of AKI because of the small number of total patients.

In conclusion, using the creatinine criteria for the definition of AKI recently proposed by the AKIN group in a large cohort of critically ill trauma patients, we found the incidence of early AKI was 6% and that its development was independently associated with a significantly higher 30-day mortality rate. The incidence of AKI after traumatic injury was less than previously reported despite the high severity of injury. One possible explanation is that rapid referral to a dedicated trauma center with highly protocolized resuscitation strategies may reduce the development of AKI. However, other factors including age and underlying health status could be responsible for this finding. These data provide the impetus to develop prediction models of the development of AKI in trauma patients in order to devise intervention studies focused on lowering mortality.
